# A Pilot Study on Predicting Ocular Hypertension in Chinese Patients Undergoing Femtosecond Laser-Assisted Penetrating Keratoplasty

**DOI:** 10.7150/ijms.106586

**Published:** 2025-02-24

**Authors:** Xueqian Cao, Jin Huang, Yang Liu, Yuting Gong, Duoduo Wang, Meihuan Wang, Zhongguo Li, Junxin Ma, Linnong Wang

**Affiliations:** 1 Department of Ophthalmology, Nanjing First Hospital, Nanjing Medical University, Nanjing, China.; 2 Department of Ophthalmology, Nanjing Yuhuatai District Yuhua Community Health Service Center, Nanjing, China.; 3 Department of Ophthalmology, Nanjing Aier Eye Hospital, Nanjing, China.

**Keywords:** Predictive model, Femtosecond laser-assisted penetrating keratoplasty, Ocular hypertension, Chinese patients.

## Abstract

**Background:** Ocular hypertension (OHT) can lead to corneal transplant failure, yet no predictive model exists for OHT after femtosecond laser-assisted penetrating keratoplasty (Fs-PKP). This study aimed to develop and validate a predictive nomogram for OHT in Chinese patients undergoing Fs-PKP.

**Methods:** A retrospective cohort study at Nanjing First Hospital included 238 patients who underwent Fs-PKP and were followed up for two years. OHT was defined as postoperative intraocular pressure exceeding 21 mmHg or an increase of more than 10 mmHg from baseline. Twenty-three variables related to recipient, donor, and surgical factors were evaluated. Key predictors were identified using the least absolute shrinkage and selection operator (LASSO) method and multivariate Cox regression, leading to nomogram development and validation via 500 bootstrap resamples.

**Results:** The final nomogram included four independent predictors: prior PKP history, history of viral ocular disease, history of ocular trauma, and intraoperative plan. The model demonstrated good discrimination with a concordance index (C-index) of 0.705 (95% CI: 0.646-0.764), consistent after Bootstrap resampling (C-index: 0.705, 95% CI: 0.648-0.747). Time-dependent receiver operating characteristic analysis at 6, 12, and 24 months showed areas under the curve ranging from 0.677 to 0.756. Calibration curves indicated strong agreement between predicted and observed outcomes. Decision curve analysis demonstrated clinical usefulness across risk thresholds of 20%-70%. High-risk patients identified by the nomogram had significantly higher OHT rates than low-risk patients (P < 0.001).

**Conclusions:** The nomogram accurately predicts OHT risk following Fs-PKP in Chinese patients, aiding clinical decision-making by preoperatively identifying high-risk individuals.

## Introduction

Penetrating keratoplasty (PKP) is a widely utilized technique for treating various corneal diseases and restoring vision 1. However, ocular hypertension (OHT) remains a significant postoperative complication, with reported prevalence rates ranging from 5.5% to 68% 2-4. Moreover, sustained elevation of intraocular pressure (IOP) can lead to corneal edema, which may ultimately result in corneal graft failure 5-8. Femtosecond laser-assisted PKP (Fs-PKP) has improved outcomes compared to conventional PKP by enhancing incision precision, reducing early postoperative astigmatism, and lowering the rates of graft rejection and endothelial cell loss 9-11. Despite these advancements, OHT remains a major concern after Fs-PKP.

Previous epidemiological studies have comprehensively investigated the risk factors for developing OHT after keratoplasty from preoperative, intraoperative, and postoperative perspectives 2, 12-15. These factors include preexisting glaucoma, elevated preoperative IOP, performing keratoplasty in conjunction with intraocular lens (IOL) removal or exchange, and the presence of aphakic or pseudophakic lens status and so on 2, 12-15. However, many of these studies focus on specific aspects and often lack a comprehensive and systematic evaluation of all potential risk factors. Moreover, the applicability of these risk factors to patients undergoing Fs-PKP is uncertain. The precise cutting capability of the femtosecond laser significantly reduces the risk of anterior chamber collapse during surgery, thereby preserving the integrity of the anterior chamber angle structure. Additionally, the improved alignment between the corneal graft and the recipient bed minimizes postoperative disruption to the angle structure. These technological advancements contribute to maintaining stable aqueous outflow pathways, reducing the likelihood of intraocular pressure elevation and lowering the incidence of postoperative OHT 9-11. Additionally, patients selected for Fs-PKP often have relatively healthier ocular conditions and may come from higher socioeconomic backgrounds, which could significantly impact postoperative outcomes. Furthermore, anatomical differences in the Chinese population, including shallower anterior chamber depth, thinner corneal thickness, and flatter corneal curvature compared to Western populations, may also affect postoperative IOP control and OHT incidence. Therefore, the risk factors associated with OHT following conventional PKP may not be fully applicable to Chinese patients undergoing Fs-PKP, given the unique anatomical and procedural differences in this population. Identifying these factors is crucial for enhancing clinical outcomes and developing tailored preventive strategies, ultimately improving patient care for those undergoing Fs-PKP.

Recent research indicates that the emergence of predictive models, which integrate varied data types and employ sophisticated statistical and machine learning methods, offers significant advantages over traditional epidemiological studies by providing more comprehensive and precise risk evaluations 16-18. Historically, predictive models in the field of corneal transplantation have primarily focused on graft failure in corneal transplantation 19-21. In our previous work, we developed a predictive model for graft failure following Fs-PKP, which has proven useful in guiding preoperative risk assessments 21. However, there is currently no predictive model specifically for postoperative OHT following either Fs-PKP or conventional PKP, particularly within the context of the unique Chinese population. This gap in the literature highlights the need for a targeted predictive model to address this critical issue.

Therefore, this study aims to develop and validate a predictive model for assessing the risk of OHT following Fs-PKP in the Chinese population. By incorporating a wide range of risk factors and leveraging advanced statistical techniques, we hope to provide a valuable tool for clinicians to identify high-risk patients preoperatively and to tailor postoperative management strategies accordingly.

## Materials and methods

### Participants and study design

This retrospective cohort study, conducted in accordance with the Transparent Reporting of a Multivariable Prediction Model for Individual Prognosis or Diagnosis (TRIPOD) guidelines (Supplementary Table 1), included 274 patients who underwent Fs-PKP performed by the same ophthalmic surgeon at Nanjing First Hospital between January 2019 and June 2021. Nanjing First Hospital is a premier institution for corneal transplantation in Jiangsu Province, Eastern China, and is particularly esteemed for its proficiency in Fs-PKP procedures 21. Patients younger than 18 years, those with incomplete medical records, or those unable to comply with the follow-up schedule were excluded from the study. Additionally, for patients who underwent Fs-PKP in both eyes, only the first-operated eye was included in the analysis to avoid bias and redundancy, as both eyes typically share similar medical histories and systemic conditions. Consequently, the final cohort comprised 238 patients with 238 eyes. This study was approved by the Ethics Committee of Nanjing First Hospital (KY20240318-KS-01) and was conducted in strict accordance with the principles of the Declaration of Helsinki. Due to the retrospective nature of the study, the Ethics Committee waived the requirement for informed consent. Nonetheless, the research adhered rigorously to ethical guidelines throughout the process.

As described in our previous study 21, the Fs-PKP procedure was conducted utilizing the Wave Light Femtosecond Laser 200, which facilitates the creation of precise mushroom-shaped incisions through a 200 kHz repetition rate and pulse energies between 160 and 200 nJ. Postoperative care adhered to a standardized regimen, with all patients receiving antibiotics and corticosteroids to minimize the risk of infection and control inflammation. Pharmacological management was customized for patients with underlying conditions. For those with a history of glaucoma or OHT, preoperative strategies were implemented to ensure IOP was maintained within normal limits prior to surgery. This involved the use of topical medications or surgical interventions as required to achieve effective IOP control. Furthermore, corticosteroids were withheld for two weeks following surgery in patients with fungal keratitis to prevent exacerbation of the infection. Patients diagnosed with infectious keratitis continued on antibiotic therapy to manage ongoing infections, while those with viral infections were administered both topical and systemic antiviral agents to ensure comprehensive viral suppression.

The follow-up assessments were systematically scheduled at predefined intervals: one week before surgery, as well as at one week, one month, six months, one year, and two years after surgery 21. For patients who developed significant postoperative complications—such as persistent epithelial defects, elevated intraocular pressure, or infectious keratitis—additional follow-up evaluations were conducted at three, nine, or eighteen months to ensure comprehensive monitoring and timely management 21. Throughout all follow-up periods, IOP was closely monitored using a handheld iCare tonometer (iCare TA01i, Helsinki, Finland). Each IOP measurement was performed three times, and the average value was used to assess postoperative OHT.

### Study outcome and definitions

Based on our previous research, we identified 23 risk factors that may be associated with postoperative OHT following corneal grafts 21. These predictors are systematically categorized into three primary groups: recipients' parameters, which encompass various patient-specific factors; donors' characteristics, which include important details about the donor cornea; and surgery-related variables, which pertain to specific aspects of the surgical procedure.

The primary outcome of the study was the occurrence of OHT, defined as a postoperative IOP greater than 21 mmHg (average of three measurements) or an increase of more than 10 mmHg over the baseline IOP, regardless of whether antiglaucoma medication or surgical intervention was required 2.

### Statistical analysis

#### Data processing

Continuous variables were expressed as means ± standard deviation, while categorical variables were analyzed using chi-square tests. All statistical tests were two-sided, with a significance level set at P < 0.05. Data processing and initial statistical evaluations were performed using IBM SPSS Statistics version 22.0.

#### Establishment of the nomogram prediction model

The development of the nomogram prediction model followed a structured two-step approach. Initially, the least absolute shrinkage and selection operator (LASSO) method, implemented via the "glmnet" package in R, was utilized to identify key predictive features. These selected features were subsequently incorporated into a multivariate Cox regression analysis to determine the independent predictors of OHT. A nomogram was then constructed using the "rms" package in R, based on the significant predictors identified in the Cox regression analysis.

#### Assessment of the nomogram prediction model

Given the limitations of a small sample size and the absence of external validation, Bootstrap resampling with 500 iterations was employed to ensure the robustness and generalizability of the model, particularly in the receiver operating characteristic (ROC) curve analysis. Time-dependent ROC analyses at 6, 12, and 24 months were performed using the "time ROC" package in R to assess the model's performance across different time intervals. The model's discriminative ability was quantified by the C-index, and calibration curves were generated using the "rms" package to evaluate the accuracy of the predictions. The clinical utility of the nomogram was assessed through decision curve analysis (DCA), implemented via the "dca.R" package. Survival curves were plotted using Kaplan-Meier survival analysis and compared using the log-rank test, facilitated by the "survminer" and "survival" packages in R. Specific analyses related to LASSO, the nomogram, ROC curves, C-index, Survival curve and DCA were conducted using R version 3.2.4.

## Results

### Demographic baseline characteristics

**Figure 1:**
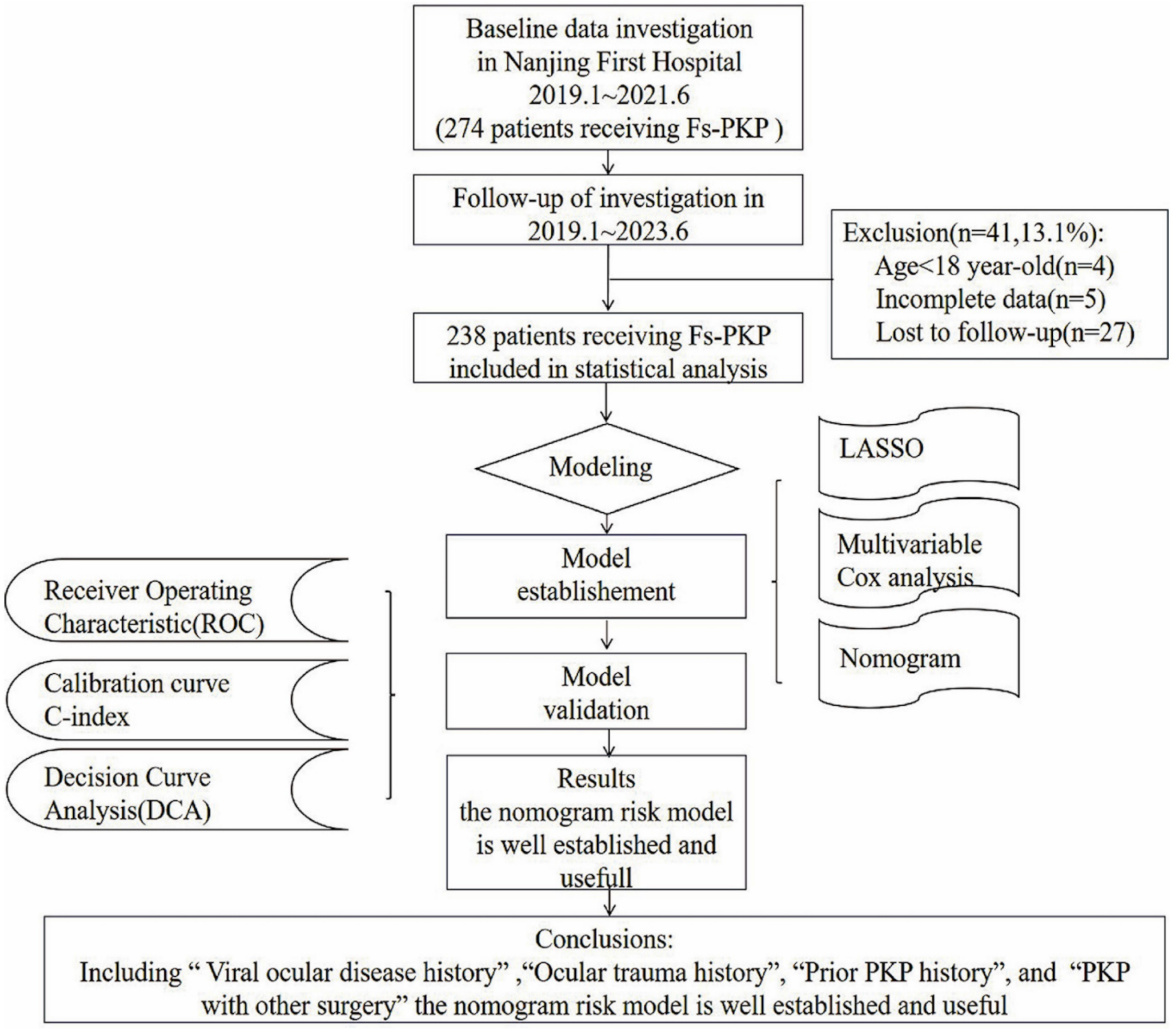
Enrollment process of this study.

As illustrated in Figure 1, which outlines the study enrollment process, 274 participants were initially considered; however, 4 were excluded for being under 18, 5 for having incomplete records, and 27 for failing to comply with follow-up. Additionally, for patients who underwent Fs-PKP in both eyes, only the first-operated eye was included in the analysis. This resulted in a final cohort of 238 patients (238 eyes). The demographic and baseline characteristics of these patients are summarized in Table 1.

### LASSO and multivariate Cox regression results

**Figure 2:**
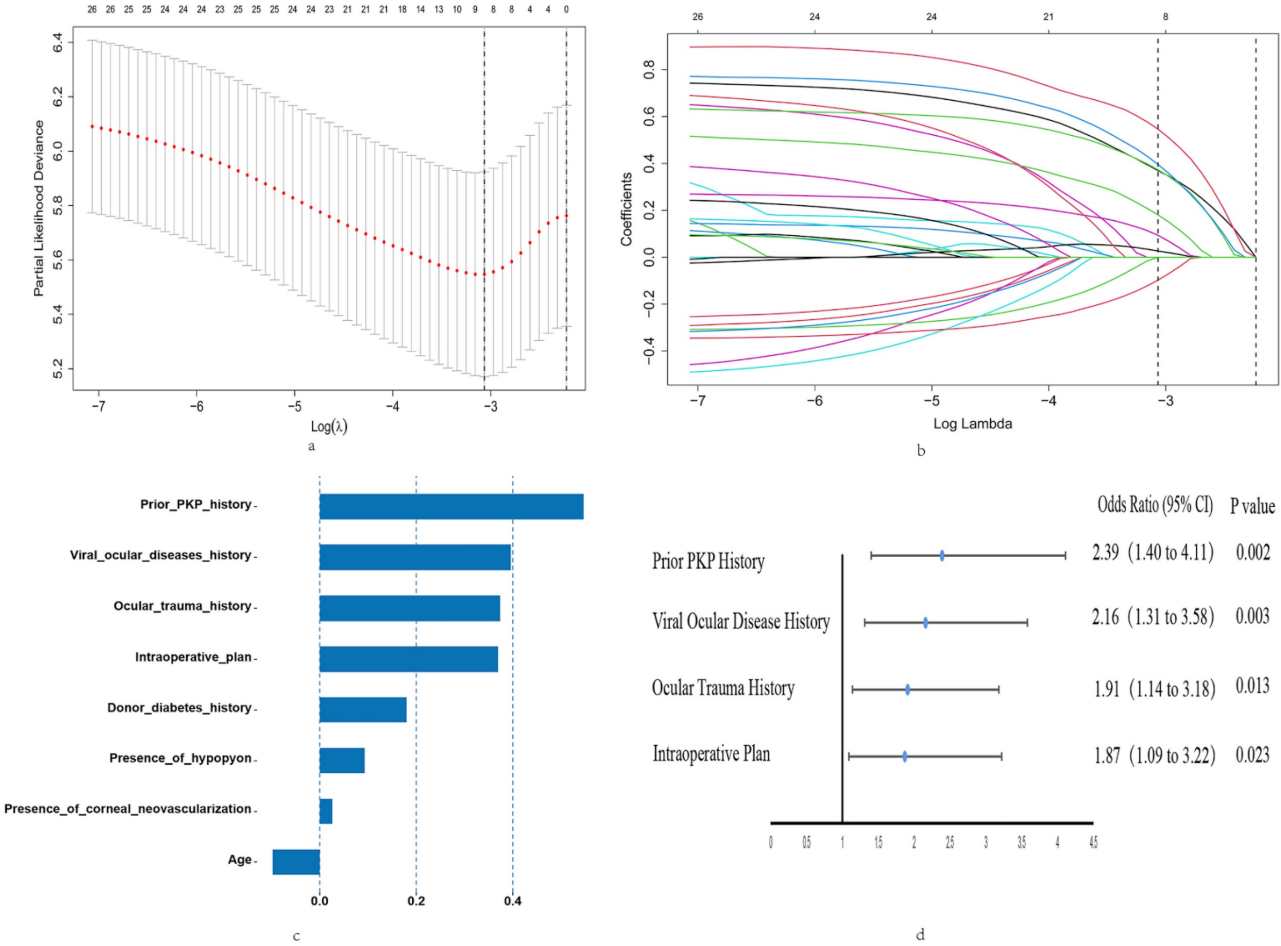
** Results of LASSO and Multivariate Cox Regression Analyses.** (a) LASSO Regression Cross-Validation Plot: Mean-Squared Error (Y-axis) versus log penalty coefficient (log λ) on the X-axis. Numbers atop the X-axis indicate the number of variables remaining at each λ value. (b) LASSO Regression Path Plot: Regression coefficients of each variable (Y-axis) against log λ (X-axis). Top X-axis values show the number of remaining variables as λ increases. (c) Eight Risk Factors Identified by LASSO: Regression coefficients (X-axis) for each of the eight risk factors (Y-axis). (d) Four Independent Predictors from Multivariate Cox Regression: Highlights the four significant predictors identified in the multivariate Cox analysis.

The LASSO method identified eight risk factors with substantial predictive value for our model. These risk factors are prior PKP history, viral ocular disease history, ocular trauma history, intraoperative plan, donor diabetes history, presence of hypopyon, presence of corneal neovascularization, and patient age (Figure 2c). Incorporating these predictors into the model significantly reduced the mean-squared error, enhancing the model's accuracy (Figure 2a). The LASSO linear regression model further validated the significance of these factors, with each demonstrating non-zero coefficients, thus confirming their statistical relevance in outcome prediction (Figure 2b).

Multivariate Cox regression analysis refined the predictive model, identifying four independent predictors for OHT occurrence after Fs-PKP: prior PKP history (OR=2.39, 95% CI (1.40, 4.11), P=0.002), viral ocular disease history (OR=2.16, 95% CI (1.31, 3.58), P=0.003), ocular trauma history (OR=1.91, 95% CI (1.14, 3.18), P=0.013), and the intraoperative plan (OR=1.87, 95% CI (1.09, 3.22), P=0.023) (Figure 2d).

### Probability of OHT after Fs-PKP

**Figure 3:**
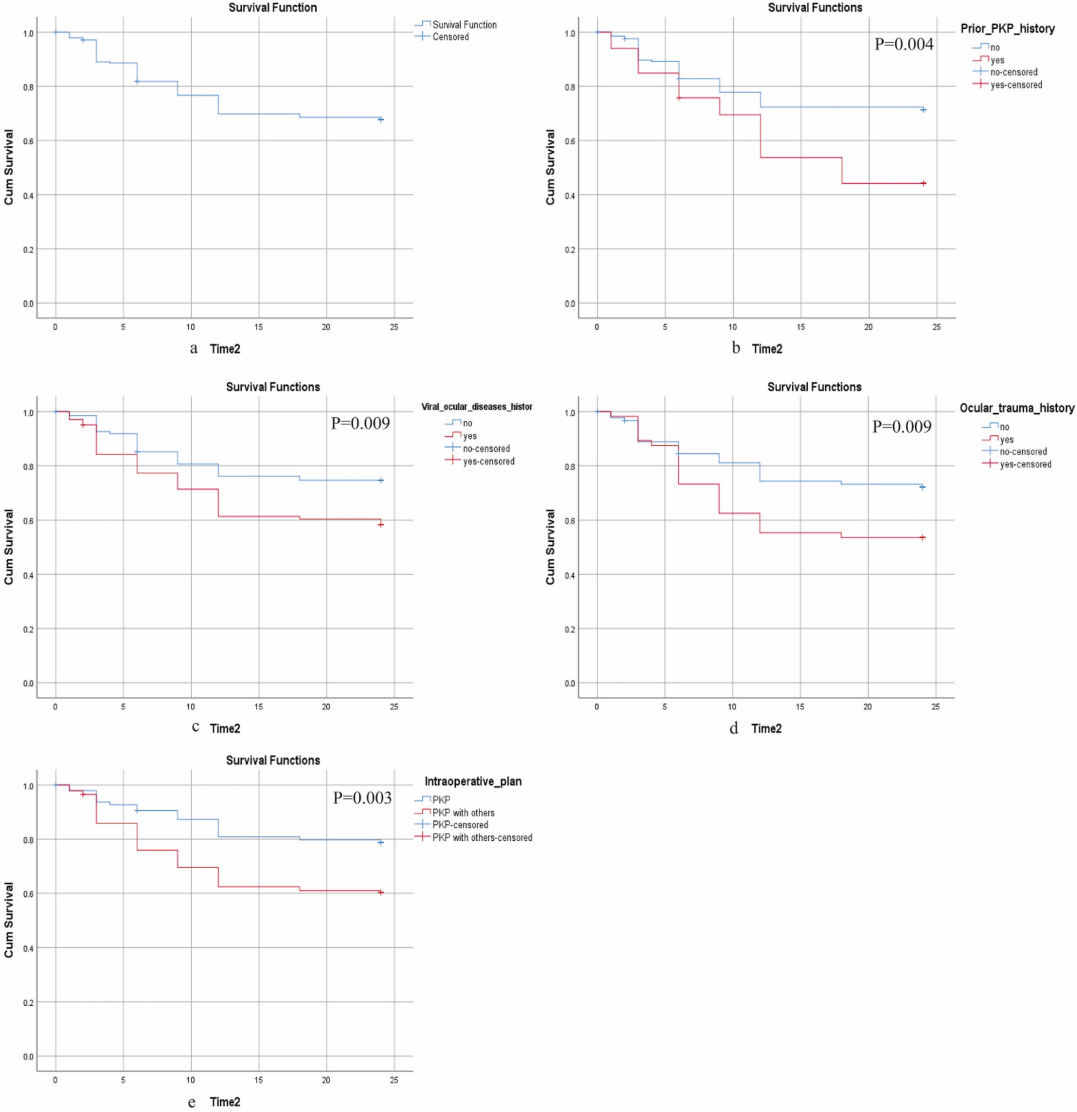
** Probability of OHT after Fs-PKP.** (a) Overall OHT rate after Fs-PKP. (b-e) Stratified OHT rate by prior PKP history (b), viral ocular disease history (c), ocular trauma history (d), and the intraoperative plan (e).

Figure 3 illustrated the cumulative probabilities of developing OHT at various time points after Fs-PKP based on the dataset: 18.07% OHT rate at 6 months (43/238), 31.09% at 12 months (74/238), and 31.93% at 24 months (76/238). Stratified log-rank tests were conducted on independent prognostic factors from Cox regression analysis to evaluate OHT rate differences (Figure 3b-f). Significant variations in OS were observed in relation to prior PKP history (P=0.004), viral ocular disease history (P=0.009), ocular trauma history (P=0.009), and the intraoperative plan (P=0.003).

### Construction and Validation of the Nomogram

**Figure 4:**
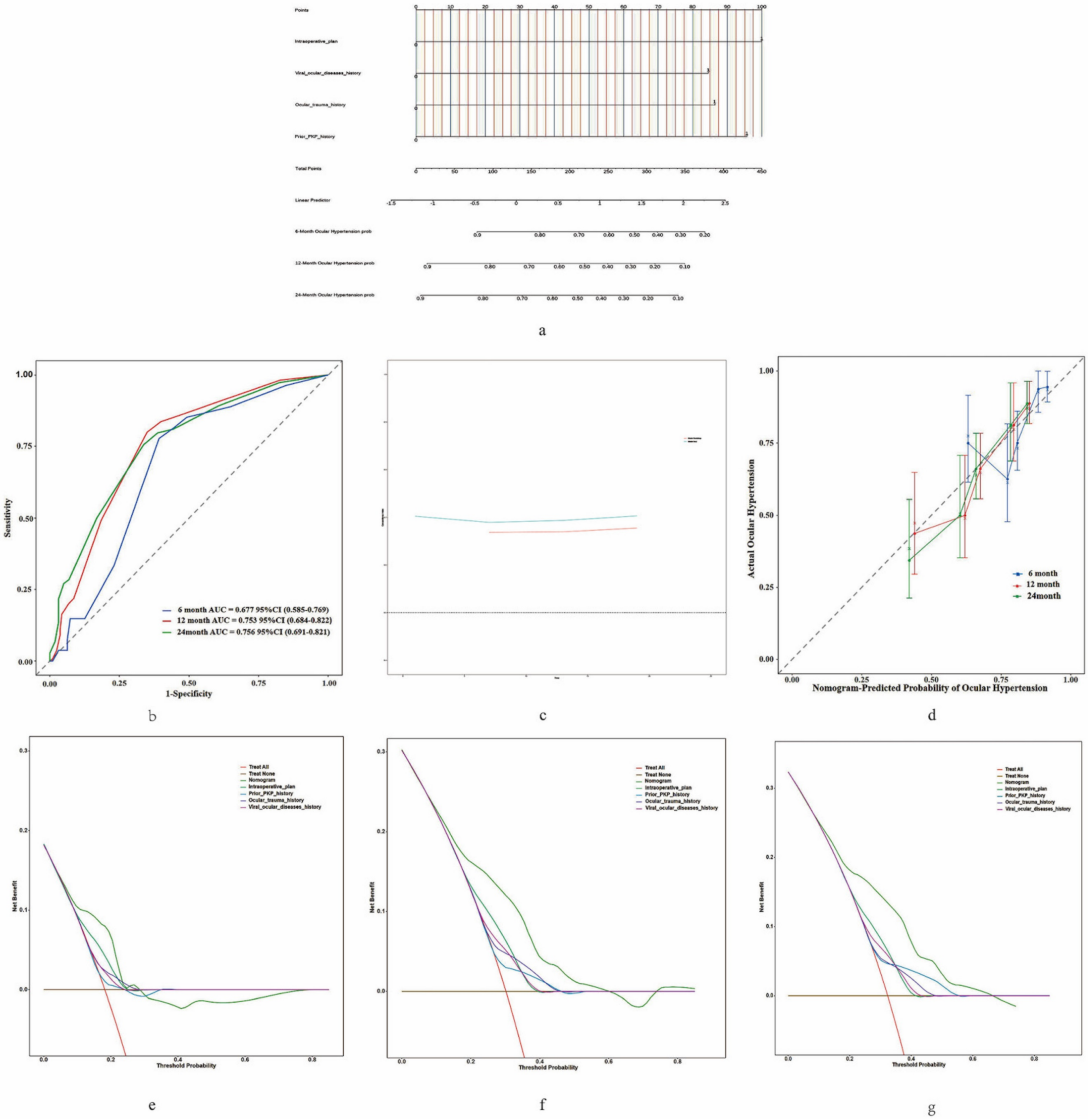
** Construction and Validation of Nomogram for OHT in Fs-PKP Recipients.** (a) Nomogram predicting the probability of OHT occurrence at 6, 12, and 24 months post-Fs-PKP, with the X-axis representing the total score based on patient characteristics and risk factors, and the Y-axis indicating the predicted OHT probability; (b) Time-dependent ROC curves, where the X-axis represents 1 - Specificity and the Y-axis represents Sensitivity; (c) C-index evaluation of the nomogram compared with 500 Bootstrap resampling, with the X-axis denoting evaluation methods and the Y-axis displaying C-index values; (d) Calibration curve plotting predicted probability of OHT (X-axis) versus actual observed probability of OHT (Y-axis); (e-f) Decision curve analyses for 6, 12, and 24 months, where the X-axis represents threshold probability and the Y-axis denotes Net Benefit.

We constructed a predictive model (Figure 4a) to estimate the risk of OHT following FS-PKP surgery, utilizing multivariate cox regression results. Time-dependent ROC analysis at 6, 12, and 24 months showed the area under curve (AUC) values ranging from 0.677 to 0.756 (Figure 4b), which were corroborated by the bootstrap ROC results of 0.648 to 0.747. The nomogram demonstrated a C-index of 0.705 (95% CI: 0.646-0.764), which remained consistent at 0.705 (95% CI: 0.648-0.747) after 500 bootstrap resampling, underscoring its reliability (Figure 4c). Calibration curves (Figure 4d) validated the nomogram's precision in predicting clinical outcomes. DCA demonstrated that the model provided optimal net benefit within risk thresholds of 20% - 70%, showing greater clinical advantage when all predictive factors were incorporated, compared to individual factors (Figure 4e-f).

### Risk stratifcation based on the nomogram

To assess the nomogram's ability to stratify Fs-PKP patients into risk groups, we calculated total points for each patient and used the X-tile program to find an optimal cutoff of 151.4. Patients were then divided into low- and high-risk groups. Kaplan-Meier analysis revealed that the high-risk group had a significantly higher OHT rate (P<0.001; Figure 3), confirming the nomogram's effectiveness in distinguishing risk levels.

## Discussion

In this study, we have innovatively developed and validated a nomogram to predict the risk of OHT in Chinese patients after Fs-PKP. The model integrates essential factors, including prior PKP history, viral ocular disease history, ocular trauma history, and intraoperative planning. By encompassing these pivotal variables, our nomogram offers clinicians a valuable and comprehensive tool to assess and mitigate the risk of postoperative OHT, ultimately improving patient care and surgical outcomes.

OHT or glaucoma is a common complication following corneal transplantation, with reported global prevalence rates ranging from 5.5% to 68% 22, 23. This considerable variability is largely due to the lack of standardized diagnostic criteria. A major challenge in defining postoperative glaucoma lies in the difficulty of assessing visual field and optic nerve changes after surgery. To address these challenges, our study focused on examining the prevalence of OHT after Fs-PKP. One of the primary difficulties in defining OHT following Fs-PKP is the inconsistency that can arise from using different techniques to measure IOP. Although Goldmann applanation tonometry (GAT) is generally regarded as the gold standard for IOP assessment 24, its accuracy is reduced in eyes with altered corneal structures. Factors such as increased corneal thickness, pronounced astigmatism, irregular corneal surfaces, abnormal corneal rigidity, and modifications at the graft-host interface can all undermine the precision of GAT measurements 25. Given these limitations, we opted to use the iCare tonometer for IOP assessment in our study. Prior research has shown that iCare achieves acceptable agreement with GAT in both normal eyes and those with post-PKP corneal edema, while demonstrating less correlation with central corneal thickness and corneal curvature. This indicates that iCare may provide a more reliable IOP measurement in post-PKP patients, making it an appropriate tool for our study 24.

Moreover, a meta-analysis involving 27,146 corneal transplant patients highlighted the diversity in IOP measurement methods used postoperatively. Although various instruments such as GAT, Tono-Pen, Mackay-Marg electronic applanation tonometer, and pneumotonometry were employed, most studies did not specify the measurement techniques utilized. Despite this variability, these studies consistently defined OHT as a postoperative IOP exceeding 21 mmHg or an increase of more than 10 mmHg from baseline 26. Considering this consensus and the limitations of other approaches, we adopted this definition in our study. By utilizing iCare for IOP assessment and adhering to this standardized definition, our findings revealed that the prevalence rate of OHT in Chinese patients undergoing Fs-PKP was 18.07% at 6 months, 31.09% at 12 months, and 31.93% at 24 months. This study provides a more accurate assessment of OHT prevalence rate in this specific patient population, considering the unique factors associated with Fs-PKP and the Asian demographic, thereby offering a valuable contribution to the field and addressing the limitations of previous research.

Prior research has largely linked post-keratoplasty OHT to intraoperative alterations, such as the deformation of the anterior chamber angle and the collapse of the trabecular meshwork 25. These alterations frequently result from the loss of structural support from Descemet's membrane, culminating in heightened outflow resistance and increased IOP 26. Although Fs-PKP is considered to have a lower likelihood of altering the anterior chamber morphology intraoperatively 9-11, our investigation still identified several risk factors, including a history of prior PKP and ocular trauma, and specific intraoperative strategies, which correspond to this mechanism. A history of PKP may compromise the structural integrity of the anterior chamber, leading to mechanical instability and subsequent angle changes. Ocular trauma has the potential to inflict damage on the trabecular meshwork and create peripheral anterior synechiae, thereby aggravating these structural alterations. Furthermore, combining PKP with other surgical interventions can profoundly impact the anterior chamber's configuration, elevating the likelihood of post-keratoplasty OHT. In addition to these mechanical factors, viral infections such as cytomegalovirus and herpes simplex virus have been associated with postoperative ocular hypertension through pathways involving inflammation and possible damage to the trabecular meshwork 27. These viruses can persist latently in the trabecular meshwork, leading to ongoing inflammation and elevated IOP. Despite administering both topical and systemic antiviral treatments to patients with a known history of these viral infections, as mandated by ethical considerations, we still found a significant association between these viral infections and OHT. This emphasizes the refractory nature of viral-induced OHT and highlights the challenges in managing such cases. Our findings reinforce the conventional understanding that the anatomy of the anterior segment and underlying primary conditions are pivotal in the etiology of post-keratoplasty OHT.

Despite previous literature emphasizing preoperative glaucoma as a significant risk factor for postoperative OHT following corneal transplantation, our study presents a different perspective 25. In our cohort of patients undergoing Fs-PKP, the majority were preoperatively diagnosed with corneal leukoma (83 out of 238) and corneal ulcers (117 out of 238), conditions that are not typically linked to a history of elevated IOP. Previous literature has often highlighted iridocorneal endothelial (ICE) syndrome as a condition associated with preoperative glaucoma, suggesting a significant risk for postoperative OHT 2. However, we consider ICE syndrome to be more suitably treated with endothelial keratoplasty rather than Fs-PKP. As a result, only 10 patients in our study had a documented history of glaucoma. Moreover, in line with ethical considerations and to ensure surgical safety, we prioritized controlling IOP to within normal ranges for all patients before they underwent Fs-PKP. These factors may help explain why our study did not find a significant correlation between preoperative glaucoma and the occurrence of postoperative OHT, offering a different perspective from previous studies.

In conventional PKP, anterior chamber hypopyon and corneal neovascularization are frequently reported as risk factors for postoperative OHT 2. Our study initially identified these factors as significant through LASSO regression analysis. However, considering the generally healthier ocular conditions of Fs-PKP patients and the administration of appropriate treatments, these factors may not independently contribute to OHT when evaluated alongside other variables. Additionally, younger patients have been reported to be more susceptible to developing OHT postoperatively, likely due to their heightened sensitivity to corticosteroids, a common cause of elevated IOP after corneal surgery 2. In our study, while age under 65 years was significant in LASSO regression analysis, it did not emerge as an independent predictor of OHT when other variables were considered. Furthermore, large graft sizes have also been reported as a risk factor for elevated IOP following surgery 2. However, it is important to note that due to the relatively small number of patients with large graft sizes in our cohort, this factor was not selected in the LASSO regression analysis. These findings suggest that the common risk factors for OHT in conventional PKP, such as anterior chamber hypopyon, corneal neovascularization, younger age, and large graft sizes, may not play the same role in Fs-PKP. This underscores a critical difference between conventional PKP and Fs-PKP and offers a more refined understanding of OHT risk factors specific to Fs-PKP.

Nomograms offer significant advantages over traditional epidemiological methods by integrating multiple predictors into a unified, individualized risk assessment model, enabling more precise and personalized predictions crucial for clinical decision-making 16-18. Historically, nomograms for keratoplasty have predominantly targeted graft failure as the primary outcome 19-21, whereas our nomogram specifically addresses the incidence of postoperative OHT. By incorporating variables, our nomogram markedly enhances the accuracy and reliability. One of the key advantages of our nomogram is its ability to provide preoperative risk stratification. By calculating cutoff values, we were able to categorize patients into distinct risk groups. Our findings demonstrated that patients in the high-risk group were more likely to develop OHT postoperatively, underscoring the clinical utility of our nomogram in identifying patients who may require closer monitoring and early intervention. Several methodological strengths further bolster the effectiveness of our nomogram. The use of the LASSO model for variable selection prior to performing multivariate Cox regression effectively reduces multicollinearity among variables, enhancing the interpretability and robustness of our findings. Additionally, applying bootstrap resampling techniques mitigates limitations associated with small sample sizes. Through 500 bootstrap iterations, we ensured that our model's predictive performance remained robust and consistent, as evidenced by the stable C-index. This rigorous approach improves the generalizability of our findings to a broader patient population. Calibration curves confirm the precision of our predictive model in forecasting clinical outcomes. DCA indicates that the model offers optimal net benefit within risk thresholds of 20%-70%, demonstrating greater clinical advantage when all predictive factors are considered together rather than individually. This facilitates more accurate risk stratification and personalized treatment plans, ultimately improving patient outcomes.

While our study has several strengths, certain limitations need to be acknowledged, along with corresponding recommendations for future research. First, the early phase of Fs-PKP adoption in China limited the number of eligible patients and the follow-up duration, resulting in a two-year observation period. While this relatively short timeframe may not fully capture long-term outcomes, our findings suggest minimal variation in the incidence of OHT between one and two years postoperatively, indicating relative stability of the anterior chamber angle during this period. Thus, the two-year follow-up provides a reasonable observation window for this study. Future studies should extend follow-up periods to evaluate long-term efficacy and identify late-onset complications. Second, the single-center nature of our study could introduce biases related to specific clinical protocols and patient demographics, potentially restricting the generalizability of our findings to other geographic and clinical contexts. Multicenter studies are needed to validate these results across diverse populations and clinical settings. Finally, our study did not leverage advanced technologies such as deep learning and artificial intelligence, which could enhance decision-making in postoperative IOP management. Future work could develop web-based nomograms for real-time personalized risk assessment and treatment recommendations. Machine learning could analyze large datasets to better identify high-risk patients and optimize interventions, while deep learning could integrate imaging data (e.g., anterior chamber angle and corneal topography) with clinical metrics to improve prediction accuracy. By addressing these limitations through extended follow-ups, multicenter studies, and advanced analytical tools, future research can refine OHT management strategies for Fs-PKP patients.

In conclusion, our study introduces a novel nomogram for predicting OHT risk after Fs-PKP in Chinese patients. By incorporating key factors like prior PKP, viral ocular disease, ocular trauma, and intraoperative strategies, this model improves the accuracy of OHT risk assessment and offers a valuable tool for clinical decision-making.

## Supplementary Material

Supplementary table.

## Figures and Tables

**Figure 5:**
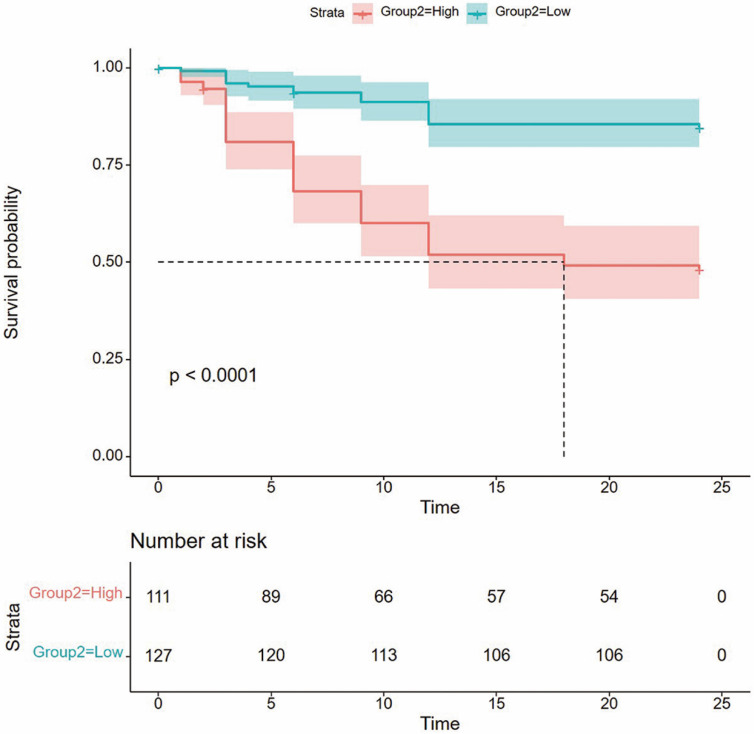
** Kaplan-Meier curve for OHT rate based on the prediction of nomogram.** Low risk, Total points <151.4 for OHT rate; High risk, Total points ≥151.4 for OHT rate.

**Table 1:** Baseline characteristics of patients undergoing Femtosecond Penetrating Keratoplasty

Characteristics	Number (%)
**Recipients' Parameters:**	
Gender (male)	142 (59.66)
Age (year) (≥65 year-old)	120 (50.42)
Diabetes history (yes, %)	26 (10.92)
Systemic autoimmune disorders history (yes)	12 (5.04)
Glaucoma history (yes)	10 (4.20)
Smoking history (yes)	47 (19.75)
Operative eye (left)	115 (48.32)
Diagnosis of ocular disease:	
corneal leukoplakia	83 (34.87)
corneal ulcer	117 (49.16)
Keratoconus	11 (4.62)
bullous keratopathy	15 (6.30)
Others	12 (5.04)
Viral ocular diseases history (yes)	102 (42.86)
Ocular trauma history (yes)	56 (23.53)
Prior PKP history (yes)	33 (13.87)
Presence of corneal neovascularization (yes)	50 (21.01)
Presence of hypopyon (yes)	57 (23.95)
Active ocular microbial infection status (yes)	116 (48.74)
**Donors' characteristics:**	
Donor age (≥60 year-old)	156 (65.55)
Donor diabetes history (yes)	40 (16.81)
**Mortality cause of donor:**	
Not cancer	161 (67.65)
Cancer	77 (32.35)
**Donor cornea's endothelial cell density:**	
<2500	156 (65.55)
>=2500	82 (34.45)
**Death-to-preservation time of donor cornea:**	
<4h	225 (94.54)
>=4h	13 (5.46)
**Storage duration of donor cornea:**	
<7 days	168 (70.59)
7-14 days	57 (23.95)
>14 days	13 (5.46)
**Reservation technique of donor cornea:**	
liquid nitrogen	12 (5.04)
Eusol C	226 (94.96)
**Surgery-related variables:**	
Corneal graft diameter (>=8mm)	69 (28.99)
**Intraoperative plan:**	
PKP without other surgery	96 (40.34)
PKP with other surgery	142 (59.66)
